# Assessment of the feasibility of high-concentration capsaicin patches in the pain unit of a tertiary hospital for a population of mixed refractory peripheral neuropathic pain syndromes in Non-diabetic patients

**DOI:** 10.1186/1471-2253-14-120

**Published:** 2014-12-15

**Authors:** Marc Giménez-Milà, Sebastián Videla, Marco-Antonio Navarro, Adela Faulí, Antonio Ojeda, Ana Bogdanovich, Luis-Alfonso Moreno, Clara Hernández-Cera, Carme Busquets

**Affiliations:** Pain Unit, Department of Anesthesiology, Hospital Clínic de Barcelona, Universitat de Barcelona, Carrer Villarroel 170, Barcelona, 08036 Spain; Catalan Society of Pain, Barcelona, Spain; Department of Experimental and Health Sciences. Faculty of Health and Life Sciences, Universitat Pompeu Fabra, Barcelona, Spain

**Keywords:** Peripheral neuropathic pain, Capsaicin patch, Clinical practice

## Abstract

**Background:**

High-concentration-capsaicin-patches (Qutenza®) have been put on the market as a treatment for peripheral neuropathic pain. A minimum infrastructure and a determinate skill set for its application are required. Our aim was to assess the feasibility of treatment with high-concentration-capsaicin-patches in clinical practice in a variety of refractory peripheral neuropathic pain syndromes in non-diabetic patients.

**Methods:**

Observational, prospective, single-center study of patients attended to in the Pain Unit of a tertiary hospital, ≥18 year-old non-responders to multimodal analgesia of both genders. The feasibility for the application of capsaicin patch in clinical practice was evaluated by means of the number of patients controlled per day when this one was applied and by means of the times used for patch application.

**Results:**

Between October 2010 and September 2011, 20 consecutive non-diabetic patients (7 males, 13 females) with different diagnoses of refractory peripheral neuropathic pain syndromes, with a median (range) age of 60 (33–88) years-old were treated with a single patch application. The median (range) number of patients monitored per day was not modified when the capsaicin patch was applied [27 (26–29)] in comparison with it was not applied [28 (26–30)]. The median (range) total time to determine and mark the painful area was 9 (6–15) minutes and of patch application was 60 (58–65) minutes. No important adverse reactions were observed.

**Conclusion:**

High-concentration-capsaicin-patch treatment was feasible in our unit for the treatment of a population with refractory peripheral neuropathic pain. The routine of our unit was not affected by its use.

## Background

Neuropathic pain management often involves the use of many therapeutic approaches (anti-epileptics, opioids, tricyclic antidepressants as well as noradrenaline and serotonin re-uptake inhibitors) that are not free of side effects [1–7]. In 2010, a new treatment (high-concentration capsaicin patches, Qutenza®) for non-diabetic peripheral neuropathic pain in adults was put on the market. It was available in our hospital from October of that same year.

High-concentration capsaicin patches should be applied by a physician or by a health care professional under the supervision of a physician. Furthermore, specific skills and procedures should be applied according to the summary of the product [8]. They include as pre-treating the area with a topical anesthetic prior to its application to reduce application related discomfort. This implies that solely applying the capsaicin patch requires an organizational infrastructure in the pain unit. In addition, within the context of the economic crisis, knowing whether this treatment can be conducted successfully in our facilities and what it implies may aid us in deciding if it should be included as an analgesic in our hospital. Therefore, the aim of this study was to assess the feasibility of the capsaicin patch applied in a pain unit of a tertiary hospital for peripheral neuropathic pain in non-diabetic patients who are non-responders to multimodal analgesia. Data on the tolerability, safety and effectiveness was also gathered.

## Methods

### Study design

This was an observational, prospective, single-center study of patients treated with the capsaicin patch in the Pain Unit. This study was based on the clinical practice without any modification. That is, the patients were administered the patches regardless of whether the study taking place. Therefore, this study simply took the opportunity to study their effects. This study was performed according to the stipulations of the Declaration of Helsinki and the level of protection of confidentiality concerning the protection of personal data as required by Spanish laws (LOPD 15/1999) was ensured. All patients gave their written informed consent for their medical information to be used for purposes of scientific research in accordance with the ethical committee of the participating site (Hospital Clinic de Barcelona). The Ethical Committee approved the informed consent in September 2010.

The capsaicin patch was applied following the instructions of the summary of product characteristics [8]. Moreover, blood pressure was measured with pressure cuffs (Critikon, GE Healthcare United kingdom, SA), every 10 minutes, from 15 minutes before applying the patch until 30 minutes after removing the patch and an electrocardiogram register was performed before and after patch application.

### Study population

Patients included in this study had to fulfill the following eligibility criteria: men or women, ≥18 years old with peripheral neuropathic pain, non-responders to multimodal analgesia and candidates to be treated with the capsaicin patch. No minimum VAS was required for inclusion. Patients with hypersensitivity to the active substance or to any of the excipients were not included.

The following data were gathered: age, sex, diagnosis, previous analgesic treatments, pain intensity, localization and area of pain, concomitant pain medication, indicators of feasibility, health state and adverse events related to the capsaicin patch as well as the effectiveness of the capsaicin patch.

### Feasibility for the application of capsaicin patch in clinical practice

The indicators used to assess the feasibility in clinical practice were “number of patients visited per day when the patch was applied” and “number of patients visited the same day of the previous week if the patch was not applied in the Pain Unit”. Likewise, as indicator of feasibility was used the times for patch application: “time to determine and mark the painful area”, “time of patch application” and “time to monitor”.

The “number of the patients visited per day when the patch was applied” was defined as the total number of patients visited in the Pain Unit the day that capsaicin patch was applied and corrected by the number of doctors who attended patients that day. The “number the patients visited the same day of the previous week if the patch was not applied in the Pain Unit” was defined as the number of patients attended to in the Pain Unit the same day of the previous week that the capsaicin patch was applied and corrected by the number of doctors who attended patients that day. The previous week was chosen whenever no patient was treated with capsaicin patch.

“Time to determine and mark the painful area” on the skin was defined as the total time (in minutes) needed by the physician to determine and mark the painful area. This variable includes: hair removal, washing and drying the skin, topical anaesthetic pretreatment and cutting the patch to match the size and shape of the treatment area. “Time of patch application” was defined as the total time (in minutes) from when the patch was applied until it was removed. “Time to monitor” was defined as the total time in minutes of monitored blood pressure from when the patient laid down on the couch until he/she was discharged from the unit.

### Tolerability and effectiveness

The tolerability and safety of capsaicin patch (adverse events related to capsaicin patch application, electrocardiogram and blood pressure during treatment application) was gathered. Likewise, the pain intensity and its evolution (at 2 and 12 weeks after the treatment) was evaluated by means of a Numerical Rating Pain Scale (NRPS, ‘0’ for no pain during the last 24 hours, and ‘10’ for the worst possible pain during the last 24 hours) at baseline (before applying the capsaicin patch treatment) and 12 weeks later (last visit). Furthermore, the pain intensity was also gathered by means of a verbal NRPS through a telephone control 2 weeks after applying the capsaicin patch. Likewise, the neuropathic pain was diagnosed by the validated Spanish versions of the DN4 with a score equal to or above 4 and by the Brief Pain Inventory (BPI). The health state was measured by means of the validated Spanish version of the EuroQol-5D (descriptive and analogic) at baseline and 12 weeks later. Concomitant pain medications during the 12-week study were recorded.

### Statistical analysis

No formal sample size was performed. The sample size was defined as the whole population treated with the capsaicin patch in the first year available in our hospital.

A descriptive analysis was performed to describe baseline population characteristics. Continuous variables were described as median (range: minimum-maximum) and categorical data were summarized as absolute frequency and percentages. As an exploratory analysis, the primary variable: the number of patients monitored per day in the Pain Unit when the capsaicin patch was or when was not applied was compared. The median and mean percentage changes in NPRS scores from baseline to 2 and 12 weeks later were calculated. The treatment success (responder) of the capsaicin patch was defined as a reduction of greater than 30% in pain intensity at 12 weeks from baseline. The percentage of responders and its corresponding 95% confidence intervals (95% CI) were calculated. The correlation between pain intensity evolution and health state was assessed. Data was recorded and analyzed using Microsoft Excel running on Windows XP (Redmont, CA).

## Results

### Patient characteristics

A total of 20 consecutive patients (7 men and 13 women), who fulfilled the inclusion and exclusion criteria, were treated with the high-concentration capsaicin patch between October 2010 and September 2011 (both included) in its first year of availability in our hospital. All these patients were included in this study and all of them gave their consent. The median (range) age was 60 (33–88) years-old. The median (range) of the history of pain treated with the patch was of 4 (0.6-14) years. The baseline characteristics of each patient included is shown in Table 1.

**Table 1:** **Baseline characteristics of all patients included (n = 20)**

	Pain background (years) ^a^	DN4	Previous analgesic treatments	Concomitant analgesic treatments during 12 weeks study
Post-herpetic neuralgia	2	9	Opioids, TL^b^, NSAIDs, anticonvulsants, antidepressants and It^d^	Opioids, TL, anticonvulsants and antidepressants
	2	8	Opioids, TL, anticonvulsants, antidepressants and IT	Opioids, TL, anticonvulsants and antidepressants,
	2	7	Anticonvulsants, Opioids, TL, antidepressants and IT	Anticonvulsants
	24	4	Anticonvulsants TL, and IT	Anticonvulsants, Opioids and antidepressant
	5	8	Opioids, TL, LI^c^ and IT	No treatment
	6	5	Opioids, TL, NSAIDs, anticonvulsants and IT	Opioids and anticonvulsants
Total knee arthroplasty post-surgical pain	4	6	TL, NSAIDs, anticonvulsants and IT	Antidepressant and Opioids
	2	5	Opioids, TL, anticonvulsants and IT	Opioids and antidepressants
	4	6	LI^,^ NSAIDs and IT	Opioids anticonvulsants and TL
Painful scar	0.6	6	Opioids, TL, anticonvulsants and IT	Opioids, TL and anticonvulsants
	3	9	TL and Opioids	TL and NSAIDs
	2	9	Opioids, TL, NSAIDs and anticonvulsants	Opioids and anticonvulsants
	4	9	Opioids, anticonvulsants and IT	Opioids, anticonvulsants, and TL
Femoral cutaneous neuropathy	14	9	TL, NSAIDs and IT	TL
	6	9	Opioids and anticonvulsants	Antidepressant
Neuroma	5	9	TL and IT	No treatment
	5	9	Anticonvulsants TL, and IT	Anticonvulsants and NSAIDs
	10	9	NSAIDs, anticonvulsants , IT, LI	No treatment
CRPS-I	1	9	TL and IT	Anticonvulsants and antidepressants
HIV-associated neuropathy	5	9	Anticonvulsants Opioids, TL, and antidepressants	Anticonvulsants

CRPS-I: complex regional pain syndrome type I.

^a^Pain background: time (years) of pain related to the process studied.

^b^Topical lidocaine.

^c^Lidocaine infusion.

^d^Interventional technique: neuroaxial blocks, intercostals blocks and sympathetic blocks.

Prior to applying the capsaicin patch treatment, the diagnosis of peripheral neuropathic pain in non-diabetic patients was confirmed: post-herpetic neuralgia (6 patients), total knee arthroplasty post-surgical pain (3), painful scar (4: axilla, foot, shoulder and post-nephrectomy), femoral cutaneous neuropathy (2: in one patient with medical history of big hematoma in the leg), neuroma (3), complex regional pain syndrome (CRPS)-I after Colles’ fracture (1) and HIV-associated neuropathy 1). The median (range) DN4 score was 6 (4–10).

All patients included had been treated with multimodal analgesia (including interventional techniques in some cases). Previous multimodal analgesia is shown in Table 1. The concomitant analgesic treatments during the 12 week study were unchanged, that is, neither the doses nor the posology was increased.

### Feasibility for the application of capsaicin patch in clinical practice

The capsaicin patch was applied to 20 consecutive patients without problems who were candidates for treatment (applicability 100%).

The median number of patients monitored per day was not modified when the capsaicin patch was applied [27 (26–29)] in comparison with when the capsaicin patch was not applied [28 (26–30)]. The frequency for patients treated with the capsaicin patch was more or less every 2 weeks and only 2 patients were treated in the same week on only one occasion. Only in one case two patient were treated in two consecutive weeks and in the same day of the week, being the reference day the two same days of the previous week (two different weeks) when capsaicin was not applied.

The median (range) total time to determine, mark the painful area and apply local anesthetic was 9 [6–15] minutes. The median (range) total time of patch application was 60 (58–65) minutes. The median (range) time of blood pressure monitoring (“time to monitor”) was 105 (99–109) minutes: 15 (14–18) minutes before applying the patch treatment and 29 (25–34) minutes for afterwards.

### Tolerability and effectiveness

#### Tolerability and safety

Pain during the capsaicin patch application (15 patients) was the most frequently reported adverse event. The pain intensity (NRPS) scored between 1 and 3. Only six (30%) patients required the administration of oral NSAIDs. Erythema on the area where the capsaicin patch was applied was reported in 9 (45%) patients, and ice was administered to 4 (20%) patients to reduce the sensation of heat. Pruritus was reported in 1 (5%) patient.

No alteration in the electrocardiogram register after capsaicin patch application was found. A slight increase in blood pressure (median of 4 mmHg in mean arterial pressure) was observed in the first fifteen minutes of capsaicin patch application. This increment in blood pressure was neither statistically nor clinically significant.

#### Effectiveness of the capsaicin patch

All patients were pre-treated with a topical anesthetic cream (EMLA, Astrazeneca Farmaceutica Spain, S.A.) prior to application of a high-concentration capsaicin patch to reduce application related discomfort. The body areas treated were: thorax (7 patients), knee (3), thigh external side (2), leg external side (1), groin (1), foot (2), left flank (1), finger (2) and wrist (1).

**Figure 1:**
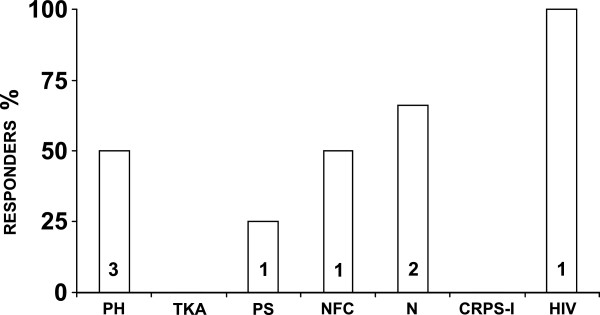
**Responders to the capsaicin patch treatment by peripheral neuropathic pain diagnosis [PH: post-herpetic neuralgia (n: 6); TKA: total knee arthroplasty post-surgical pain (n: 3); PS: painful scar (n:4); NFC: Femoral cutaneous neuropathy (n: 2); N: neuroma (n: 3); CRPS-I: complex regional pain syndrome type I (n: 1); HIV: HIV-associated neuropathy (n: 1)]**.

**Figure 2:**
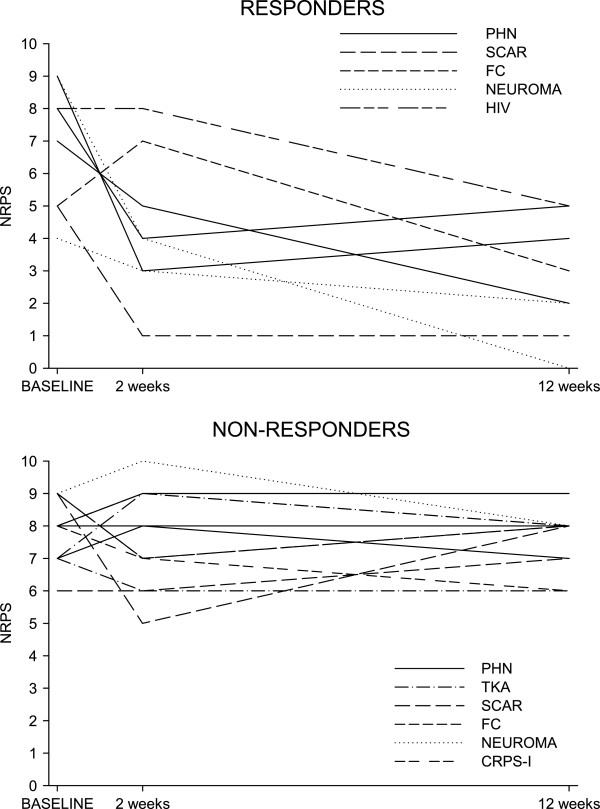
**Evolution of pain according with responders and non-responders.**

The median (range) pain intensity at baseline was 8 (4–9) [mean (sd): 7.5 (1.7)] and it was 7 (0–9) [mean (sd): 5.8 (2.6)] at 12 weeks from the capsaicin patch treatment. The overall median (range) percentage of the reduction in pain scores after 12 weeks was 13% (−17%, 100 [mean (sd): 23% (32)]. Eight (40%, 95% CI: 19%-64%) out of 20 treated patients were considered responders (≥30% of reduction from baseline): 4 (57%) out of 7 men and 4 (31%) out of 13 women. In the group of responder patients, the median (range) percentage of the reduction in NPRS pain scores was 56% (38%-100%) [mean (sd): 62.1 (21–0)], and in non-responders 11% (−17%, 25%) [mean (sd): 3.2 (13.6)]. Based on the responder definition of a pain reduction ≥50% from baseline, 5 (25%, 95% CI: 9%-49%) out of 20 patients treated with a high-concentration capsaicin patch [1 (14%) man and 4 (31%) women] were achieved a pain reduction ≥50% from baseline (Table 2). Figure 1 shows the percentage of responders for peripheral neuropathic pain diagnosis. It is worth noting that only 4 patients (20%) did not previously receive an interventional treatment. Of those four patients, 2 patients were capsaicin patch responders. The evolution of pain is depicted in Figure 2.

**Table 2:** **Health state evolution based on EuroQol-5D**

	Health state (EuroQol-5D)			
	Baseline		At 12 weeks	Difference
	Descriptive	VAS ^a^	VAS ^a^	
Post-herpetic neuralgia	11121	60	75	+15
	22233	25	50	+25
	22232	50	40	−10
	11131	30	80	+50
	22232	25	40	+15
	12222	70	50	−20
Total knee arthroplasty post-surgical pain	22232	90	20	−70
	21121	50	60	+10
	22333	30	40	+10
Painful scar	22333	20	30	+10
	11132	90	90	0
	21333	35	20	+15
	22232	20	20	0
Femoral cutaneous neuropathy	11231	80	90	+10
	11232	50	80	+30
Neuroma	21222	70	80	+10
	12232	70	40	−30
	11231	50	100	+50
CRPS-I	11323	50	50	0
HIV-associated neuropathy	21221	50	70	+20

^a^Visual Analogue Scale (VAS) of EuroQol-5D.

Concomitant pain medications during the 12 weeks of study are summarized in Table 1. No new medication was started during the 12 weeks of study. The patients carried on with the medication administered prior to capsaicin patch treatment because they presumably were on optimal doses.

The median (range) health state (EuroQol VAS) at baseline was 50 (20–90) [mean (sd): 50.8 (22.0)], and the descriptive system scored is shown in Table 1. The health state at 12 weeks was 50 (20–100) [mean (sd): 55.3 (25.0)]. Six patients (30%) had a EuroQoL VAS equal to or greater than 70, suggesting a good quality of life. In the group of responders, the EuroQol VAS at 12 weeks was a median (range) 80 (50–100) [mean (sd): 77.5 (19.2)] and it was 40 (20–80) [mean (sd): 44.2 (20.2)] in the no responder group; and the median (range) of change (baseline minus at 12 weeks) in the EuroQol VAS was of 12.5 (−20, 50) [mean (sd): 16.9 (23.7)] in responders and of 10 [−70, 30] in non-responders [mean (sd): 0.4 (27.3)].

## Discussion

This observational, prospective 12-week study reports novel data on the feasibility (single 60-minute topical application) of the high-concentration capsaicin patch in patients who did not respond to multimodal analgesia. One of the most important reasons for performing this feasibility study was to identify both real and potential problems that may be encountered in applying this new treatment (capsaicin patch) routinely in the clinical practice. Our pain unit has three treatment rooms, three consultations rooms, and we work altogether with two specialized pain nurses leading to a flexible work minimizing the negative impact of the treatment application. During the consultation clinic the physician could develop the normal activity because the nurses were trained in performing this procedure including the patient monitoring.

It is remarkable that no more than one patient was treated with a capsaicin patch per day and it was only in 1 week that the capsaicin patch treatment was applied to two patients. The aforementioned along with the small sample size may affect the interpretation of the feasibility data. However, the results suggest that the use of high-concentration capsaicin patch in our pain unit of a tertiary hospital may not have a relevant organizational impact insofar as the burden of visited patients was not affected the work-force. Thus, it can be carried out as planned within the estimated times.

The use of high-concentration capsaicin patches requires organization [8–10]. This study has allowed us, besides determining the potential impact that the treatment might have on our routine, to determine the adequacy of facilities and equipment that are presently available and how much real time is required to manage this treatment. The use of this treatment required a space where the treatment could be applied and the patient could be somewhat comfortable during the application. In addition, a nurse specialized in pain treatment is required for nearly 2 hours to apply the capsaicin patch, whose tasks are rescheduled to be performed during certain phases of the procedure, also a physician must be available at the facility.

In spite of the time required to manage this treatment, capsaicin patch application time did not affect the number of patients treated per day in our Pain Unit. The capsaicin patch was not a difficult treatment to manage and it might be an alternative in patients with peripheral neuropathic pain and multimodal analgesia non-responders.

Another objective of this feasibility study was to convince us of the treatment’s value. Conclusions on the effectiveness of capsaicin patch could not be drawn due to the study design, the low number of patients included and to the variability of indications of peripheral neuropathic pain tested. To our knowledge, limited or no evidence based on clinical trials is available on the efficacy of the capsaicin patch for peripheral neuropathic pain different from post-herpetic neuralgia and HIV-associated neuropathy in non-diabetic patients [11, 12] or in diabetic polyneuropathy [7, 12]. As expected, the group of patients diagnosed with post-herpetic neuralgia had a mean reduction of pain with respect to baseline similar to that reported in the registration clinical trials [13]. Likewise, if all patients treated with high-concentration capsaicin patch are considered, the overall mean pain reduction (23%) was also the expected. It is remarkable that 6 responder patients had been treated with interventional techniques with no relief of pain before capsaicin patch. A possible explanation would be that the action of anesthetics on depolarization is limited in time. In contrast, capsaicin causes a defunctionalization which could last up weeks until recovery [14–16]. On the other hand, no interventional technique was performed on 2 responder patients, which represent 25% of responder patients. This could open a possibility for the capsaicin patch to be a therapeutic alternative in patients that have not received interventional treatment. Nevertheless, clinical trials of efficacy are required to answer this interesting question. Nonetheless, the reduction of pain seems to be associated with a health state improvement. The patients with a higher basal quality of life (>70 VAS of EQ) are better responders. The descriptive analysis performed might suggest that the profile of responders might be related to the absence of problems with walking about, problems with self-care, no problems with the performance of usual activities and to a state of moderate anxiety or depression at the moment of capsaicin patch application. Likewise, the patients that responded to the capsaicin patch improved their quality of life.

The capsaicin patch was well tolerated. As expected, the most commonly reported adverse reactions were local transient pain at the application site, erythema and pruritus.

At this point, it is worth mentioning that in our patients topical anaesthetic pretreatment was only applied for a time less than 60 minutes as specified in the Summary of Product Characteristics. In no case was it necessary to remove the patch before time. To manage the acute pain during the application of the capsaicin patch, oral analgesics (NSAIDs) and local cooling (ice) were administered in 30% and 20% of the patients, respectively. In fact, short-acting opioids, as recommended in the summary of product characteristics, were not necessary.

This study has limitations that should be considered before drawing any conclusion. The study design (observational and without control group) is not ideal for evaluating the impact of the capsaicin patch in clinical practice or for evaluating efficacy. The small sample size could lead to underestimating or overestimating the results obtained. In fact, this study was planned as exploratory. Data concerning the time of the analgesic treatment before inclusion in the study was not collected, and this might be affected the effectiveness. The evolution of pain was only based on 3 controls (at baseline, at 2 weeks and at 12 weeks). In this vein, when the study was planned, it was thought that the moment of pain intensity control (between 8:00 am to 3:00 pm) could not be standardized. In order to minimize the effect of variability at the moment of gathering the pain intensity, this one was based on the worst pain in the last 24 hours. Likewise, in our pain unit, the interventional procedures are performed twice a week in the operating theatre but on different days from capsaicin patch application. Hence, the feasibility results may not be applicable in hospitals with different organizational structure.

## Conclusions

Data from this study provides evidence that the high-concentration capsaicin patch treatment was feasible in the pain unit of a tertiary hospital for the treatment of a variety of refractory peripheral neuropathic pain syndromes in a population of non-diabetic patients. The routine in our unit was not affected by its use. Nevertheless, pharmaco-economic studies are necessary to validate the feasibility of the capsaicin patch. No important adverse events were gathered.
